# Laboratory Evaluation of the Shinyei PPD42NS Low-Cost Particulate Matter Sensor

**DOI:** 10.1371/journal.pone.0137789

**Published:** 2015-09-14

**Authors:** Elena Austin, Igor Novosselov, Edmund Seto, Michael G. Yost

**Affiliations:** 1 Department of Environmental and Occupational Health Sciences, University of Washington, Seattle, WA, United States of America; 2 Department of Mechanical Engineering, University of Washington, Seattle, WA, United States of America; Columbia University, UNITED STATES

## Abstract

**Objective:**

Finely resolved PM2.5 exposure measurements at the level of individual participants or over a targeted geographic area can be challenging due to the cost, size and weight of the monitoring equipment. We propose re-purposing the low-cost, portable and lightweight Shinyei PPD42NS particle counter as a particle counting device. Previous field deployment of this sensor suggests that it captures trends in ambient PM2.5 concentrations, but important characteristics of the sensor response have yet to be determined. Laboratory testing was undertaken in order to characterize performance.

**Methods:**

The Shinyei sensors, in-line with a TSI Aerosol Particle Sizer (APS) model 3321, tracked particle decay within an aerosol exposure chamber. Test atmospheres were composed of monodisperse polystyrene spheres with diameters of 0.75, 1, 2 3 and 6 um as well as a polydisperse atmosphere of ASHRAE test dust #1.

**Results:**

Two-minute block averages of the sensor response provide a measurement with low random error, within sensor, for particles in the 0.75–6μm range with a limit of detection of 1 μg/m3. The response slope of the sensors is idiomatic, and each sensor requires a unique response curve. A linear model captures the sensor response for concentrations below 50 μg/m3 and for concentrations above 50 μg/m^3^ a non-linear function captures the response and saturates at 800 μg/m^3^. The Limit of Detection (LOD) is 1 μg/m^3^. The response time is on the order of minutes, making it appropriate for tracking short-term changes in concentration.

**Conclusions:**

When paired with prior evaluation, these sensors are appropriate for use as ambient particle counters for low and medium concentrations of respirable particles (< 100 ug/m^3^). Multiple sensors deployed over a spatial grid would provide valuable spatio-temporal variability in PM2.5 and could be used to validate exposure models. When paired with GPS tracking, these devices have the potential to provide time and space resolved exposure measurements for a large number of participants, thus increasing the power of a study.

## Introduction

Exposure to fine particulate matter (PM2.5) air pollution is associated with a variety of adverse health outcomes, including all-cause mortality, cardiovascular disease, cardiopulmonary disease, and lung cancer [1–9]. The health burden attributable to PM2.5 exposures is large: the most recent global assessment estimates 3.3 million deaths (7.1% of the world’s deaths in 2004) were attributable to PM2.5 exposure, including 2.5 million cardiopulmonary disease and 1.3 million ischemic heart disease deaths [10]. These estimates are higher than previous World Health Organization estimates [11], reflecting improvements in exposure assessment [12]. Even in regions of the developed world, where strong health-protective standards exist, efforts to reduce the impacts of air pollution continue. For example, in the United States, 123 counties do not meet the 24-hour PM2.5 standard [13], and short-term increases in PM2.5 levels are estimated to cause tens of thousands of excess deaths per year [7,14–16].

Efforts to understand aerosol dynamics, reduce PM2.5 concentrations in communities, inform environmental justice studies, reduce exposure assessment errors for epidemiologic studies, and facilitate new models of community-led or community-engaged research may benefit from improved understanding of the spatiotemporal distribution of PM with respect to mobile and stationary sources of particulate emissions. Due to the limitations in the coverage and density of U.S. EPA monitoring sites, various modeling approaches have been used to spatially resolve air pollution patterns in urban areas and map pollutants on finer scales [17]. An alternative to modeling that provides ongoing empirical data on air pollution concentrations at the neighborhood level, involves augmenting the existing network of EPA sites, with additional monitoring locations [18]. The cost of Federal Reference Method (FRM) continuous monitoring PM2.5 instruments, has made it infeasible to establish large dense networks of real-time FRM aerosol monitors. But, short-term monitoring studies of certain urban environments, notably immediate areas downwind of major roadways, have measured elevated concentrations of ultrafine PM, black carbon (BC), elemental carbon (EC) and metals [19]_,_ which have led to efforts to protect public health [20].

The recent availability of new low-cost optical aerosol sensors based on the principle of particle light scattering, has motivated new research to evaluate their performance characteristics. We are interested in understanding the environmental health applications in which such sensors may be useful, as well as understanding the limitations of these sensors in terms of sensitivity, upper and lower limits of detection, sensor to sensor variability, and the dynamics of their response to changing concentrations. This current study builds upon prior work in which we conducted field calibrations of a low-cost sensor, the Shinyei PPD42NS [21]. In that study, the sensor was co-located with a various commercially available particle counters as well as a Federal Equivalent Method (FEM) beta attenuation monitor. Short-term measurements indicated strong correlation between the Shinyei and particle counters costing orders of magnitude more. Long-term measurements (approximately 4 months) indicated lower, but still moderately good correlation with the FEM beta attenuation monitor. In addition, this sensor was deployed in a high concentration urban setting alongside reference optical and gravimetric methods and shown to have good correlation [22]. While these studies suggest that the sensor could be used to augment existing regulatory monitoring networks, they also raise numerous questions as to the limits of the sensor’s performance that are best answered in more controlled laboratory environments. This study presents our findings from laboratory work, evaluating the Shinyei PPD42NS for a variety of known particle compositions and concentrations.

## Methods and Materials

### The Shinyei PPD42NS

**Fig 1:**
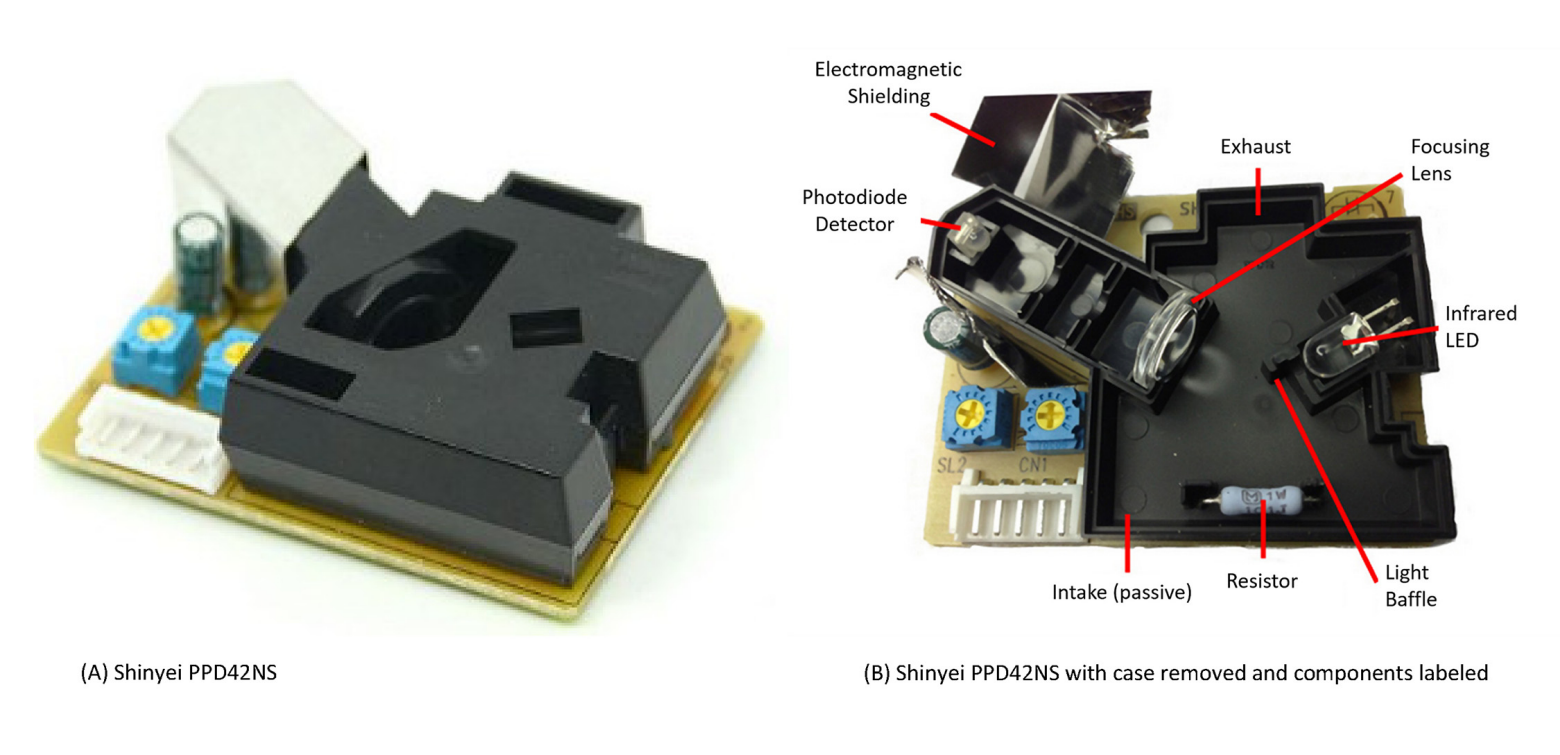
Shinyei PPD42NS. On the left is an exterior image of the sensor. On the right a view of the inside showing the positioning of the various sensor components. (A) Exterior view of sensor. (B) Interior view of sensor. This image was initially published on http://www.takingspace.org/make-your-own-aircasting-particle-monitor

The Shinyei PPD42NS is an inexpensive particle counter that costs approximately $10 in small quantities, and can be obtained easily from various electronics retailers (Fig 1). It consists of a light chamber that routes air past a light emitting diode and photo-diode detector that measures the near-forward scattering properties of particles in the air stream. A resistive heater located at the bottom inlet of the light chamber helps move air convectively from the bottom inlet to the top outlet of the chamber. Additional electronics control the underlying detection and signal processing, which results in a digital pulse width modulated output. The raw sensor signal consists of low pulse occupancy (a duration of time that digital signal is held low), which is proportional to particle count concentration. For our laboratory experiments the PPD42NS was connected to a small custom microprocessor circuit developed by our group that reads and stores the low pulse occupancy signals at 1-second intervals.

### Laboratory Chamber and Reference Instrument

The comparison instrument for this test was a TSI Aerodynamic Particle Sizer (APS) Spectrometer 3321. This instrument provides real-time size-resolved counts for particles ranging in size from 0.5–20 microns. Mass is not directly measured by this instrument. However, for polystyrene particles of known diameter, this instrument provides highly accurate count and mass data [23,24]. Particle count information is converted to mass by inputting the known density of polystyrene beads using Eq 1:

$$dM=dN\;*\frac{\pi }{6}\;D_{p}^{3}\rho$$ (1)

Where the density (ρ) is that of the material being aerosolized, the change in number concentration (dN) and the diameter of the particle (D_p_) are directly measured by the APS as aerodynamic diameter.

The inherent assumption in this calculation is that the particles are spherical which is true in the case of the polystyrene test aerosol. The number concentration of the test aerosol was maintained below 1500 #/cc to minimize the coincidence errors in the APS counts. Size distributions of the test aerosols are provided in the supporting information to demonstrate the monodisperse properties of the aerosol generated. We tested four different PPD42NS sensors over the course of this lab study. The sensors were labeled 1 through 4 and were tested in pairs.

**Fig 2:**
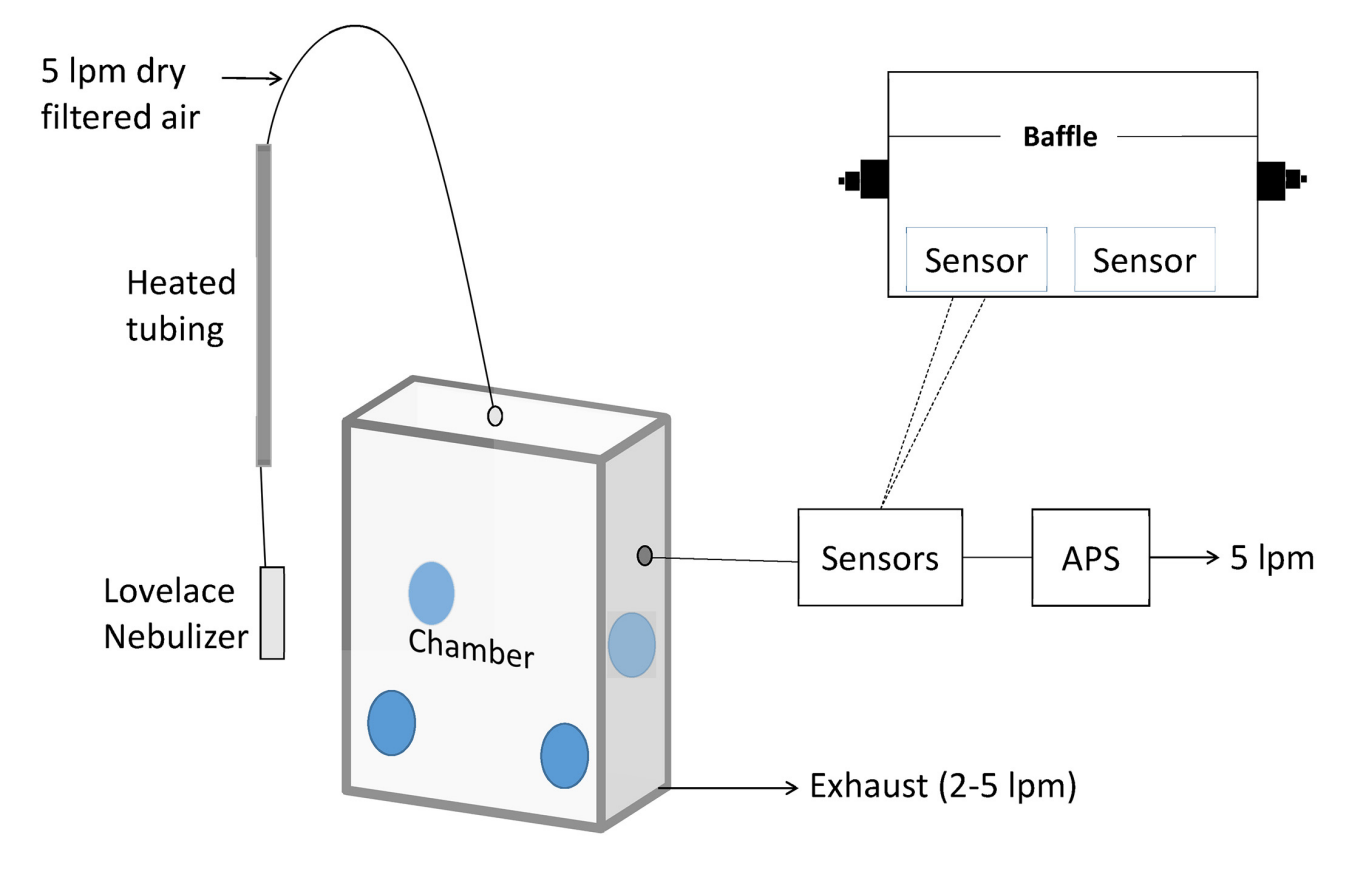
Schematic of Experimental Set-Up. The blue circles indicate the location of the mixing fans inside the chamber.

Two sensors were mounted along the interior wall of an airtight box that measured 6 x 21 x 8 cm. This box was placed downstream of the mixing chamber described in the following paragraph. The interior volume within the box was reduced to approximately 500 cm^3^ by placing a fixed baffle along the inner length of the box (Fig 2). Air was actively aspirated at 5 lpm through the sensor enclosure using the internal pump of the APS. The sensors were placed in the airtight enclosure arranged in series upstream of the APS inlet. Static-dissipative silica rubber tubing (McMaster-Carr) was used to minimize particle deposition on the inner walls of the sampling train. The sensors were provided 5V of power, as per their operating instructions, output was captured by an Arduino microcontroller and output to a laptop using a serial connection. The raw sensor output is on the 1 second reporting interval. Raw sensor output corresponds to the photodiode pulse width in a 1 s period. This is called the Lo Pulse Occupancy by the manufacturer and is purported to be proportional to PM_2.5_ mass concentration.

Two different chambers were used as part of these experiments. The first, was 0.3 m^3^ in volume and maintained a slight negative pressure through an exhaust flow. This flow was set at 5 lpm (with a tolerance of 20%). Mixing fans were located in the corners of the chamber to ensure the air was well-mixed. The second chamber was a bench-top model with an internal volume of 0.45 m^3^. Walls were acrylic and all inputs were sealed. Negative pressure was maintained through outflow to the APS and sensors (5 lpm). The same aerosol generating mechanism was used for both these chambers. The size distribution of the particles between the two chamber set-ups was very similar for the 1 μm particles (S3 Fig and S4 Fig). In addition, the half-life of the particles in each experiment was similar (25 ± 3 minutes residence time).

### Experimental approach

Test aerosols considered during this series of experiments were monodisperse 0.75, 1.0, 3.0 and 6.0 μm polystyrene microspheres in solution (Polysciences, Inc. Warrington, PA); polydisperse ASHRAE test dust #1. ASHRAE 52.1/52.2 standard test dust composed of 72% ISO 12103–1, A2 Fine Test Dust, 23% powdered carbon and 5% milled cotton linters (purchased from Air Filter Testing Laboratories, Inc. 4632 Old LaGrange Road Buckner, KY). Size distributions of this test atmosphere is provided as supporting information (S1 Fig).

Aerosol, both liquid and dry dust, was generated using a Lovelace nebulizer and introduced into the chamber using 1.5 meter, 2” diameter stainless steel tubing and mixed with a 5 lpm dry air flow to allow for drying prior to introduction. The stainless steel tubing was heated using a heated tape set to 200 C. Aerosol was generated until the concentration inside the chamber was above 1000 #/cc. The aerosol generation then was halted and the decay of the remaining particles was tracked using both the APS and the sensors in series. A small exhaust flow was maintained throughout the experiments.

### Analyses

All collected data were time-matched to the nearest second prior to analysis. The APS data were aggregated over 5-second intervals, and a spline function was used to interpolate concentrations between these 5-second time points. The Shinyei sensors collected data over 1-second intervals. After being time-matched, the data were transformed using a 2-minute moving average. Models were fit individually for each sensor because of sensor-specific responses.

The relationship between mass concentration as determined by the APS and the raw sensor readings (low pulse occupancy) from the Shinyei was described using three alternative models: a linear model, a polynomial model and a Generalized Additive Model (GAM) that incorporated semi-parametric spline terms. The number of terms to include in the polynomial model was determined based on the Bayesian information criteria (BIC). The spline model was developed using the *gam* function in R and fitted with a penalized thin-plate spline created with the *s* function.

The saturation point (upper limit of detection) of the detectors was calculated, for each particle size, as the concentration for which a 10 μg/m^3^ increase in the APS concentration resulted in less than a 0.2 reported change in Lo Pulse occupancy time on the Shinyei sensor. An alternative saturation point of the sensor response was determined using a non-linear, least squares model. The model is a von Bertalanffy growth equation with two parameters, presented as Eq 2:

$${Shinyei}_{i}=A\;*\;(1-\;e^{-K*{APS}_{i}}\;)$$ (2)

Where A is the asymptotic Shinyei value

K is the growth rate.

APS_i_ is the APS concentration at time i

Shinyei_i_ is the Shinyei value at time i

This model was fit to the data using non-linear least square estimates (nls) package in R. This model was chosen because it captured the shape of the experimental curves and it directly estimates the asymptotic growth parameter. The parameters were initialized with starting values of A = 100 and k = 0.1 and all model runs converged within 50 iterations. The estimated value of A was used to calculate the maximum detection limit (APS mass values) of the sensors for different experimental conditions.

The lower limit of detection (LOD) was calculated as 3 times the standard deviation of the sensors’ response for readings on the APS that were less than 1 μg/m^3^. To facilitate interpretability, this LOD was converted to μg/m^3^ using the response plot based on exposure to 1 μm and 3 μm diameter polystyrene.

Bland-Altman plots were used to judge the correlation between the Shinyei PM_2.5_ reading, after applying our response curve to 1 um polystyrene beads over a range of concentrations. The model applied was the penalized spline model described above. Data were fit using the *predict* function in R. These plots show the mean of the two measured values on the x-axis and the difference between the measurements on the y-axis. In addition, the Bland-Altman plots were used to contrast the performance of the two different non-linear models applied to the data.

The response times of the sensors were judged based on a step-function experiment. The air drawn through the APS and sensors alternated between room air and air from the chamber containing a 1 μm polystyrene test atmosphere.

Analysis was performed in R version 3.0.2.

## Results

**Fig 3:**
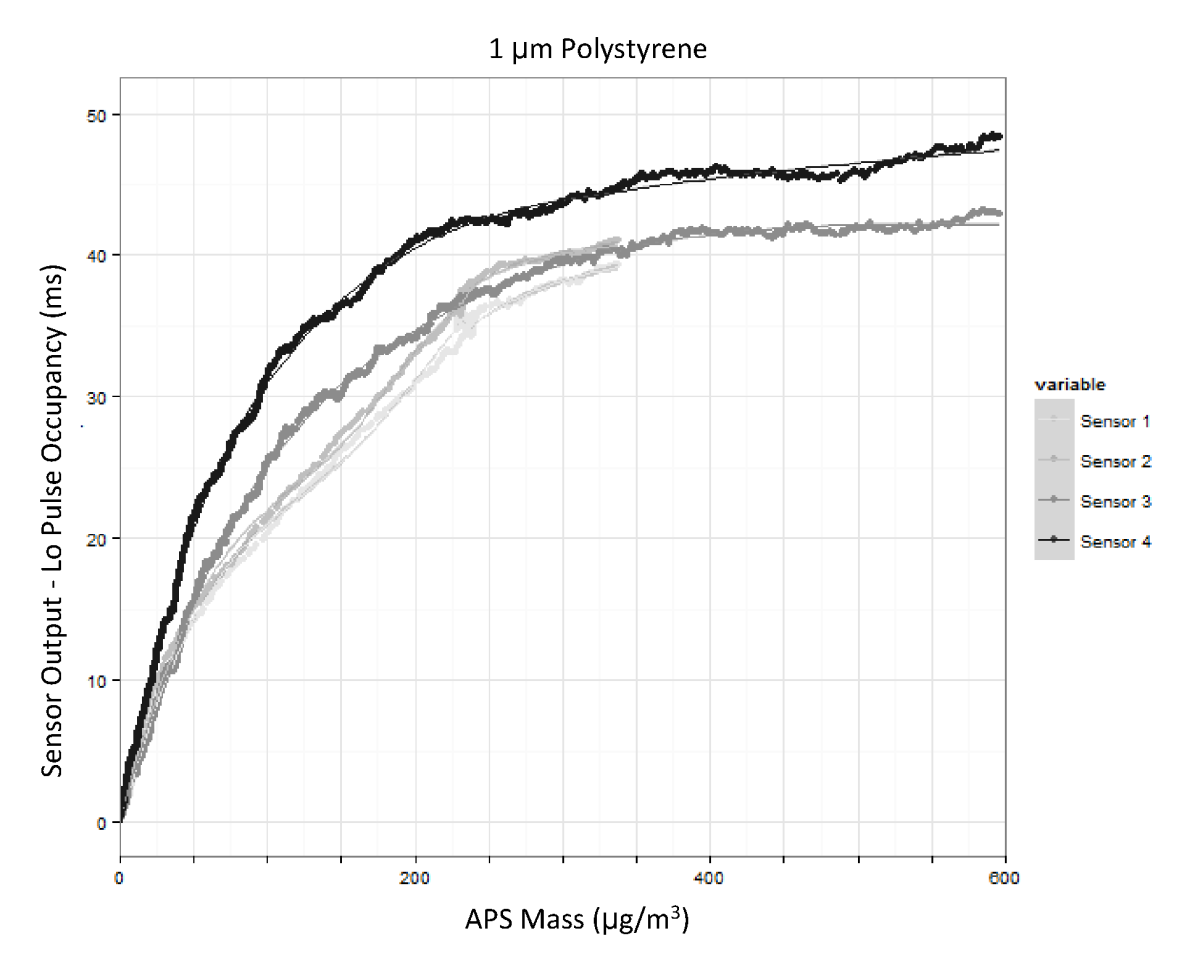
Response to a test atmosphere composed of 1 μm polystyrene beads. This figure includes raw-data and a penalized spline to describe the response shape.

**Fig 4:**
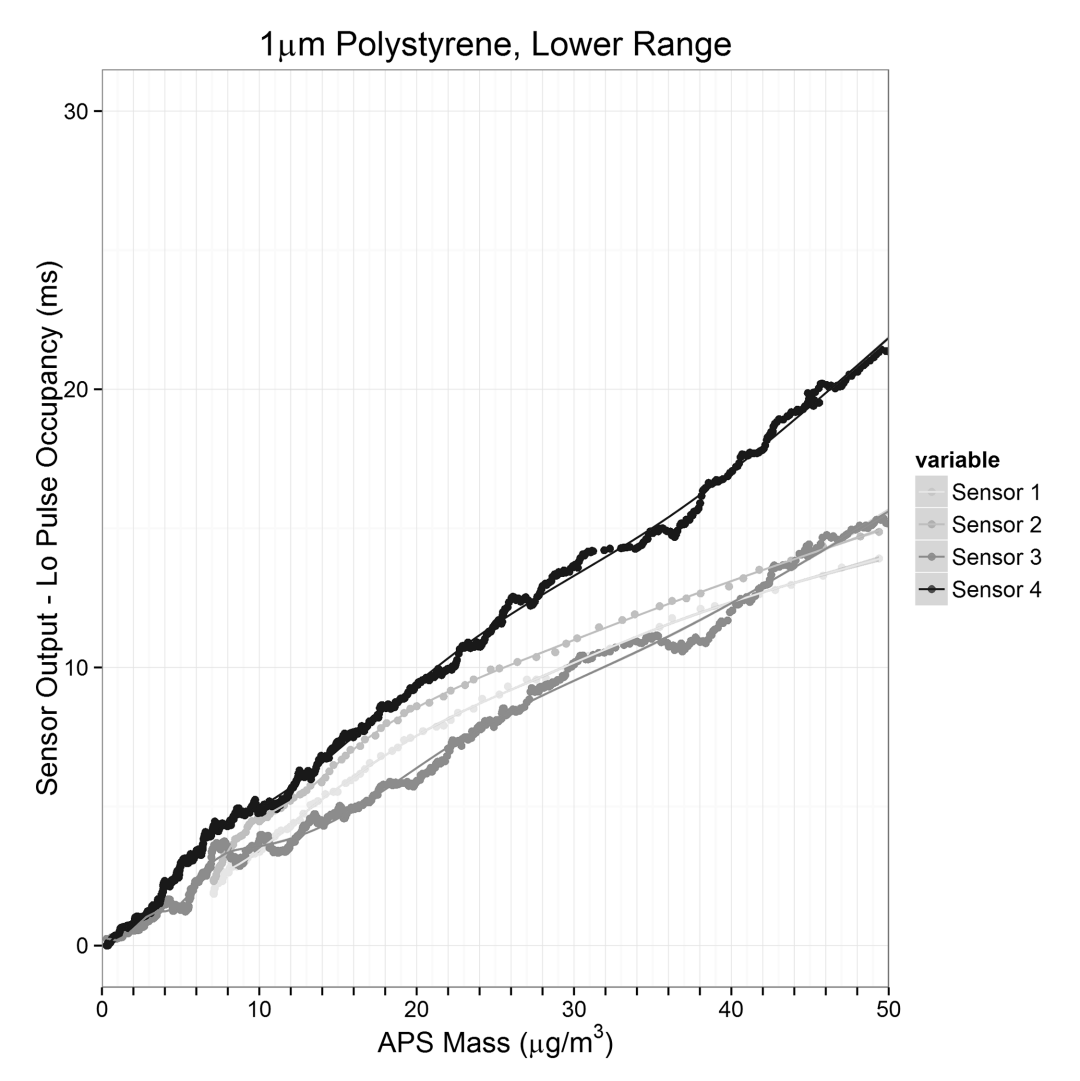
Response to low concentrations of Polystyrene.

Fig 3 shows the response of the four different sensors to a test atmosphere of 1 μm polystyrene beads. The response of the Shinyei sensors compared to the APS was non-linear over the concentration range examined. For this section, results are presented as a function of mass concentration. This is for interpretability with respect to ambient concentrations. As described above, the conversion from number concentration to mass concentration in the case of a monodisperse aerosol constitutes of a simple constant adjustment. From 0–50 μg/m^3^ the response of the Shinyei was essentially linear for all test atmospheres. Above 50 μg/m^3^ the response was attenuated. In Fig 4, the response between 0–50 μg/m^3^ is presented. On this more restricted range, the response of the Shinyei sensors is well-captured by a linear function. Table 1 shows the linear fit of two sensors to different test aerosols from 0–50 μg/m^3^. The output of Sensor 2 was not recorded (serial bus malfunction) for the 0.75 μm test atmosphere.

**Table 1:** Linear Model for concentrations below 50 ug/m3. The response of the sensors to test atmospheres is given in terms of a linear slope and error.

	Sensor 1		Sensor 2	
	Slope	adj. R2	Slope	adj. R2
**0.75 μm**	1.02 ± 0.06	0.66	-	-
**1.00 μm**	3.05 ± 0.05	0.99	2.75 ± 0.18	0.98
**2.00 μm**	5.13 ± 0.05	0.99	5.25 ± 0.03	0.99
**3.00 μm**	12.00 ± 0.17	0.99	9.93 ± 0.07	0.99
**6.00 μm**	12.43 ± 0.13	0.86	25.79 ± 0.33	0.80
**ASHRAE**	5.40 ± 0.03	0.97	8.15 ± 0.04	0.97

The LOD of the Shinyei sensor was calculated based on 348 observations for which the APS measurement was 1 μg/m^3^ or less. As with our measurements, these observations were based on 2-minute block averages of the raw output from the sensors. The standard deviation of these observations was 0.04 units for sensor 1 (n = 173) and 0.02 units for sensor 2 (n = 175). Since the Shinyei units must be converted to mass, we used the linear model developed for concentrations below 50 μg/m^3^ to determine that the LOD was:

$$LOD=3\times {SD}_{i}\times {Slope}_{i}$$ (3)

Where, SD is the standard deviation of the Shinyei readings when exposed to air with less than 1 μg/m^3^ particles

Slope_i_ is the slopes calculated in Table 1.

The LOD for 1 μm polystyrene is 1.0 μg/m^3^ and for 3 μm it is 1.9 μg/m^3^_._

**Fig 5:**
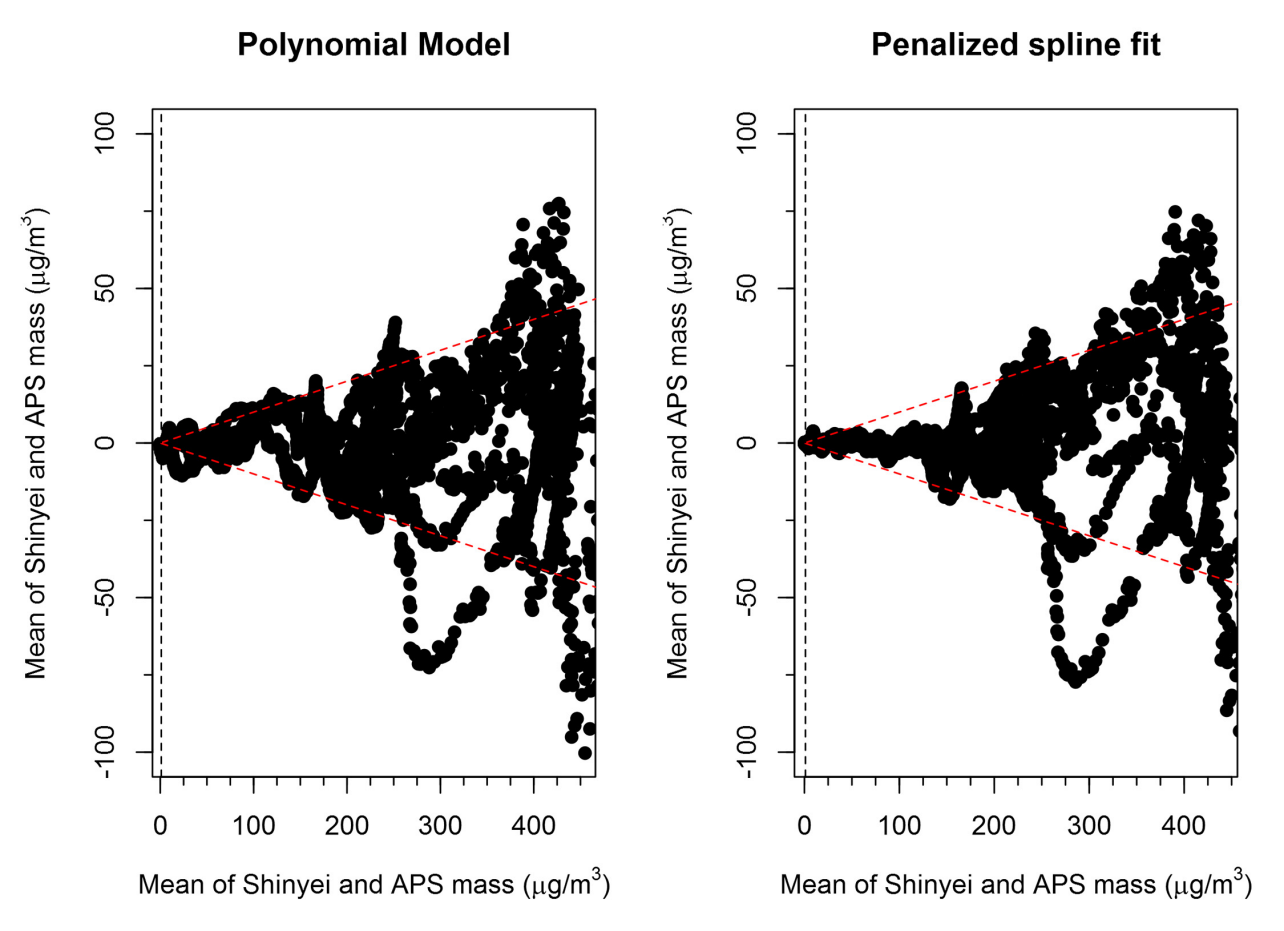
Bland Altman Plots (4 sensors pooled together). The red dashed lines represent a 10% error on the mass measurement. The black vertical dashed line represents the LOD.

In order to capture the non-linear response of the Shinyei sensor to 1 μm particles at higher concentrations two different models were tested. The first was a polynomial model with five terms. Polynomials with 2 to 6 terms were considered, but the 5 term polynomial model was deemed to have a best fit based using the BIC as a statistical criteria. The second was a semi-parametric penalized thin-plate spline model that captured the non-linear relationship between the Shinyei response and the aerosol mass concentration. The advantage of the polynomial model is that it can easily be parametrized and extrapolated. The advantage of the penalized spline model is that it uses the data variability to produce a non-linear fit that best describes the shape of the response while penalizing for overfitting. The results of the two models were compared using a Bland-Altman plot in order to select the model with the best predictive power (Fig 5). For concentrations below 150 μg/m^3^ the penalized spline models performed better than the polynomial model, with the difference between the modeled Shinyei and APS measurements consistently below 10% of the mean of the measurements. Therefore, we conclude that the penalized spline model performs better than the polynomial model, acknowledging that the response differs by sensor, and hence, the splines will be sensor-specific. Although the spline model produces a semi-parametric response, it can still be used to convert Shinyei observations to equivalent APS measurements using a prediction function implemented in R.

The von Bertalanffy growth equation (Eq 2) allowed for the estimation of the saturation point for the different experimental atmospheres. Table 2 presents these results for all polystyrene experiments. Because the ASHRAE atmosphere was not generated in concentrations high enough to estimate the maximum response, these were not included in this table.

**Table 2:** Maximum detection limit (determined by modeling sensor response).

	Sensor 1	Sensor 2
**0.75 μm**	383 μg/m3	-
**1.00 μm**	830 μg/m3	950 μg/m3
**2.00 μm**	1280 μg/m3	1180 μg/m3
**3.00 μm**	2910 μg/m3	2500 μg/m3
**6.00 μm**	68 μg/m3	25 μg/m3

**Fig 6:**
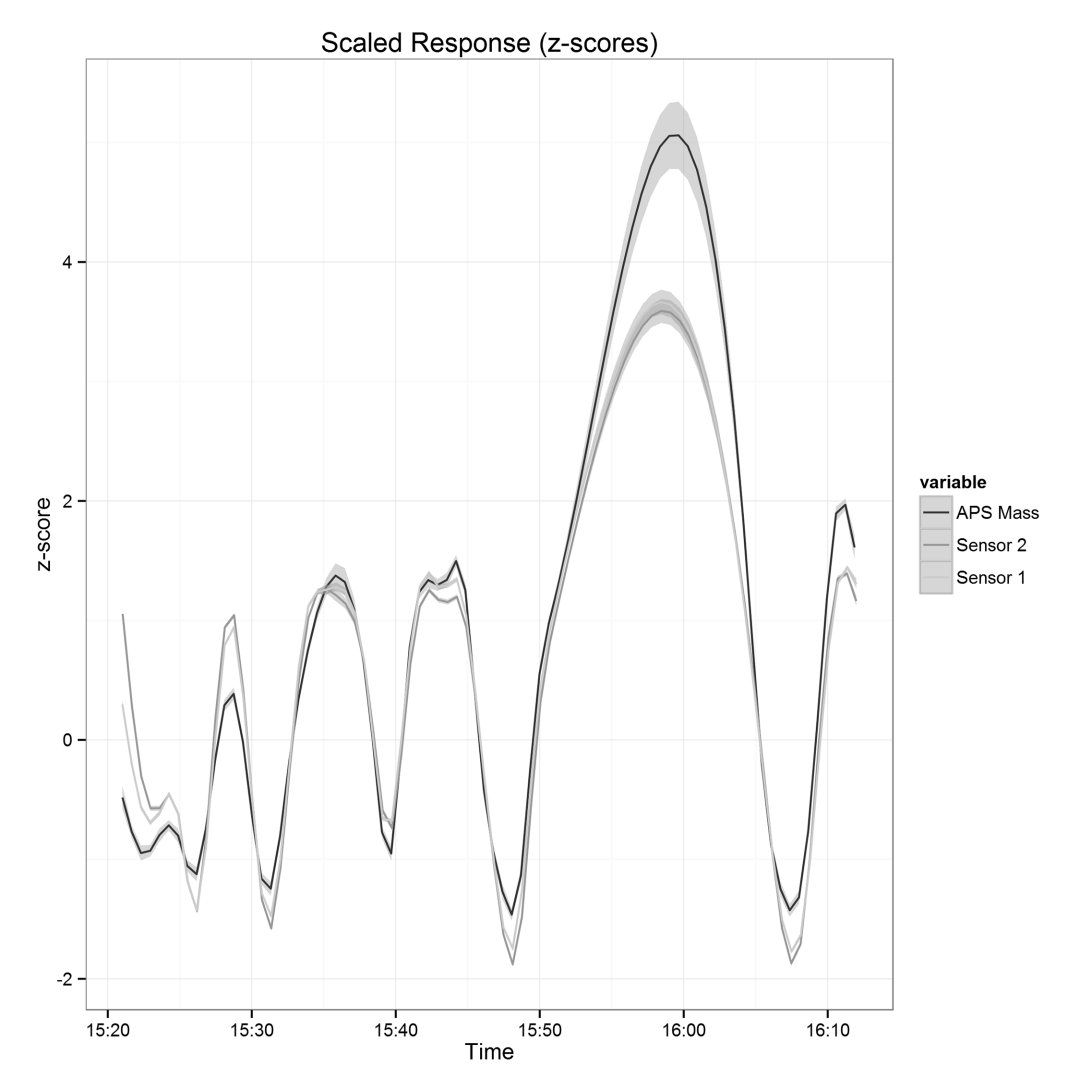
Response time of the sensors and APS to changes in concentration of a 1 μm test atmosphere.

The result of the response time experiment was presented graphically in order to judge the response time of the instruments (Fig 6). The time it took for the instruments to return to a baseline reading after the exposure was switched from polystyrene to filtered air was determined as the response time in this experiment. The mean response time, over 4 repetitions, was 3m:45s (sd 27s) for the APS, 3m:45s (sd 8s) for sensor 1 and 3m:50s (sd 1s) for sensor 2. Thus, the response time of the Shinyei is highly comparable to that of the APS, typically judged to be a fast response instrument.

## Discussion

**Fig 7:**
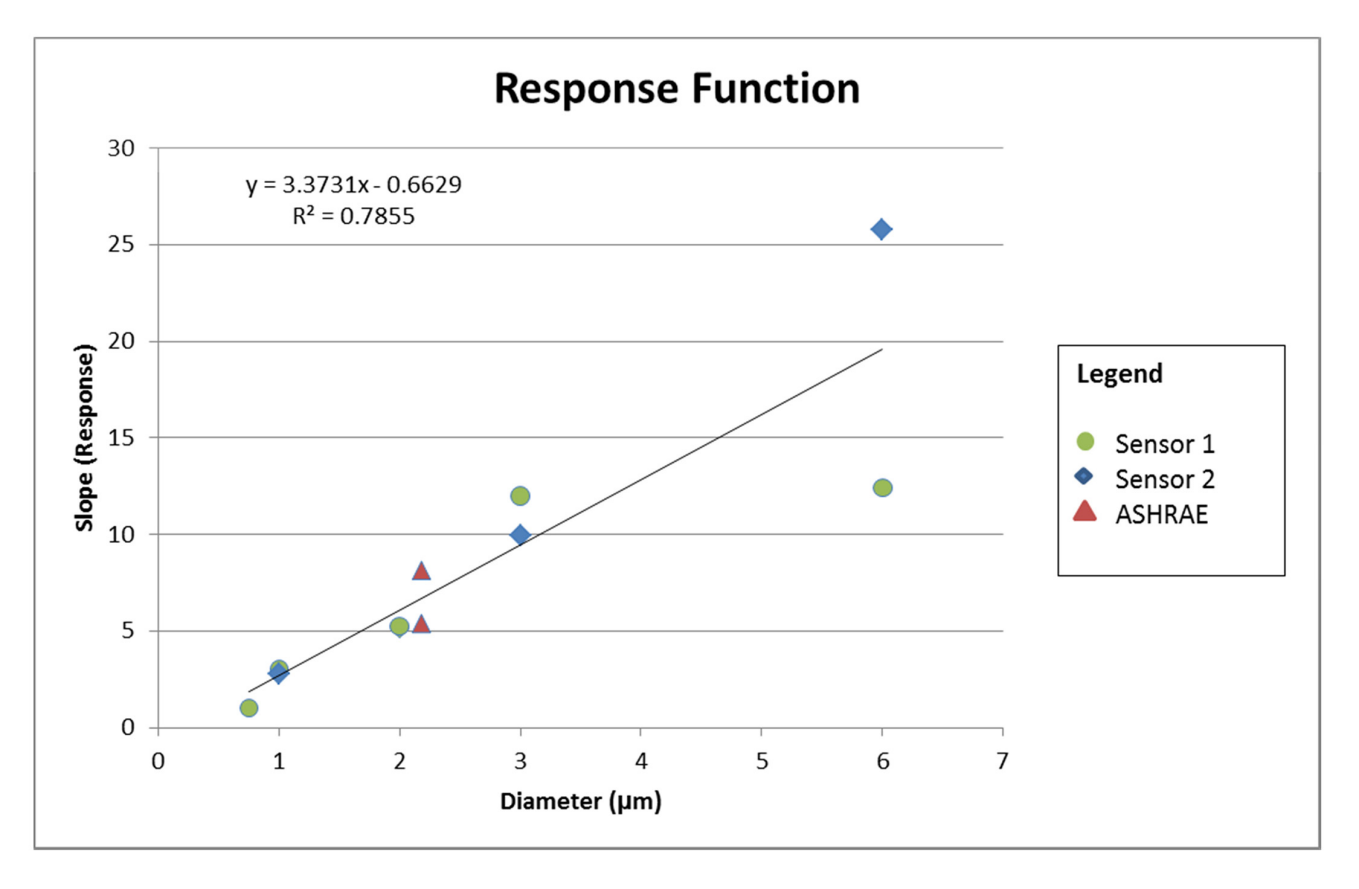
Relationship between sensor response and particle diameter.

Several questions were explored in this comparison between the Shinyei particle counter and the validated TSI APS instrument that could not be determined in field deployments. The first was the LOD of the Shinyei to a variety of particle sizes, which proves to be low enough to make this an appropriate sensor in most ambient and indoor conditions. The second was the relationship between the sensor response and particle diameter. In fact, as can be expected from an instrument relying on the principle of near-forward scattering, there is a linear relationship between particle diameter and sensor response (Fig 7). For particles under 3 um, the relationship is consistent for the different sets of experimental conditions. For particles in the 6 um diameter range, the response is consistent with the established linear trend, but clearly subject to unacceptable variability for use in field conditions. This suggests that these sensors would benefit from the addition of a size-selective inlet in order to reduce interference from particles with aerodynamic diameters greater than 2.5 μm. Fig 7 also indicates that the smallest particle detected with this sensor is 0.5 μm. We conclude that this particle sensor is best suited to detecting particles in the accumulation mode, but is not appropriate for assessing exposures to ultrafine or coarse mode particles.

This comparison also demonstrates that the precision of the Shinyei sensors, when compared to the APS is quite high with extremely small standard deviations estimated for the slope and high R^2^ (Table 1). In addition, when the results of all the experiments are pooled, the response of the instruments is highly associated with the diameter of the challenge particle (R^2^ = 0.80). The Bland-Altman plots demonstrated that when the sensors are corrected for their idiomatic response, the results agree with the APS count data within 10% for concentrations below 150 μg/m^3^. This suggests that the accuracy of the Shinyei, when the idiomatic sensor response is accounted for, is acceptable for deployment conditions.

**Fig 8:**
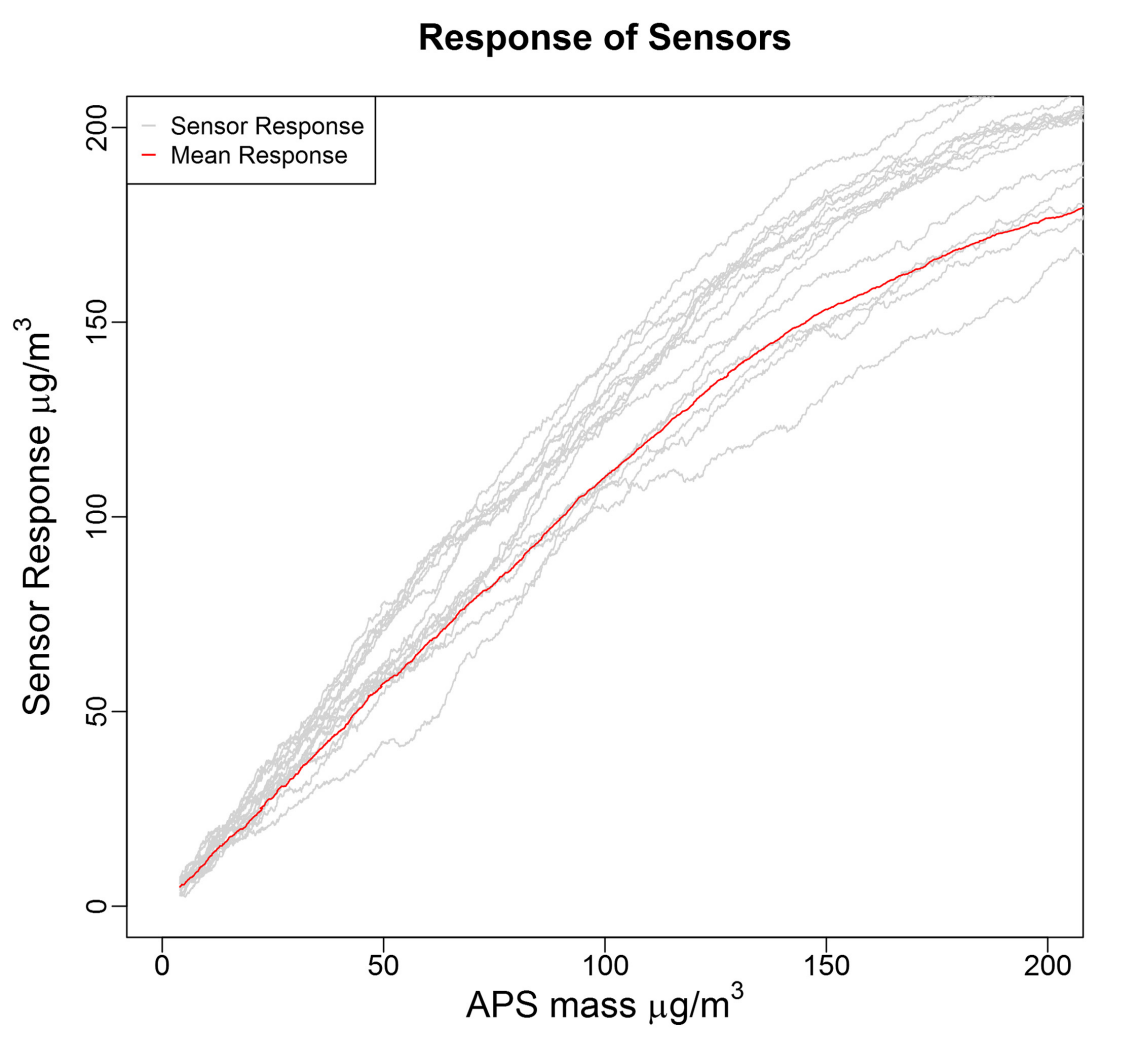
Idiomatic response of Shinyei sensors.

The Shinyei sensors can be reliably used to detect particles ranging in size from 0.5–2.5 μm within a set of specified conditions. This corresponds to particles in the respirable range as described by the EPA. The response of these sensors to generated aerosol atmospheres is idiomatic, meaning each sensor follows its own response curve. This was further confirmed by testing of a 20 additional sensors using a 2 μm polystyrene test atmosphere (Fig 8) using the same set-up described above. In addition, the accuracy of the mean response of these 20 sensors in the linear 0–50 μg/m^3^ range was judged to be 9% after converting each sensor response to a mass concentration using a linear regression, as described in the methods section. These results are presented in the supporting information (S7 Fig). Therefore, before being deployed, each sensor requires comparison, either through co-location or laboratory challenges, along the entire range expected to be encountered during sampling. This could be accomplished as we have in this study with a test chamber with monodisperse 1 μm polystyrene beads, or alternatively as we have done previously by colocation with reference instruments [21,22]. Secondly, the magnitude of the response of the sensors depends on the diameter of particles being measured. The change in slope between 1 and 3 μm polystyrene beads is consistent between sensors; however the response to larger particles is highly variable. This suggests that these sensors would benefit from the addition of a size-selective inlet in order to reduce interference from particles with aerodynamic diameters greater than 2.5 μm.

The saturation of the sensors at moderately high concentrations has relevance to their use in certain applications. For ambient monitoring within European and North American urban environments these sensors have adequate limits of detection and maximum limits of detection. However, if these monitors are to be used in environments with PM_2.5_ concentrations that routinely exceed 500 μg/m^3^, they will likely saturate and possibly not capture the true range of concentrations. Therefore, in these types of monitoring situations other sensors, or possibly dilution with a filtered air stream should be considered.

Because of the low-cost associated with these sensors, and the possibility to calibrate them for field use, they can serve as new tool to reliably obtain continuous exposure estimates over finely detailed spatial areas. The results of this paper suggest that if such sensors are deployed in communities to augment current regulatory monitoring networks, measurements are expected to be reasonably accurate and precise. However, the conversion from particle count to mass concentrations may be difficult. A possible solution would be to centrally locate a validated method such as an impactor or TEOM in order to calibrate the sensor data. Having a continuous monitoring network with high spatial coverage would have the advantage of allowing for more accurate exposure estimates. This type of coverage would also be valuable in calibrating spatio-temporal PM_2.5_ distribution models. Future work needs to be performed to assess the parameters of such a network given the inherent errors associated with individual sensors as well as inherent errors associated with the deployment of a large number of sensors over a small spatial area.

The small size, low power requirements and the ability for the Shinyei sensors to be integrated with other monitoring devices such as GPS or mobile phones, make this sensor a promising tool for personal monitoring studies, in which a study participant could wear such a monitor. The ability to obtain detailed continuous exposure information from a large number of study participants may allow for additional insight into the association between short-term exposures to respirable PM_2.5_ and their acute health effects. Such a monitor would might also provide spatial and time resolved data and allow for the determination of where and when individual participants receive the bulk of their exposure. Again, the applicability of the Shinyei to personal monitoring studies largely depends on whether the upper limit of detection might be exceeded. The monitor could be saturated in certain occupational and developing world studies.

One important limitation to integrating these sensors into larger studies at this time is that we have not evaluated their long-term performance. Possible problems that may arise are drift in the sensor’s response, as well as the sensors failing during deployment. Sensor drift might occur due to deposits forming on the lens of the optical sensor. While we do not know the length of time the sensors can perform without appreciable drift, it is possible to develop field protocols in which sensors are routinely recalibrated. This is no different from the traditional and more costly instruments that also require routine recalibration.

A possible solution to the recalibration concerns may be ongoing field co-locations of the low cost sensors with reference instruments. Routine checks for deviation over time from initial calibration curves, may indicate that it may be time to recalibrate all the deployed sensors in a network, through some combination of cleaning the lenses, replacing worn sensors (after all, they are inexpensive), and re-estimation of the individual calibration regression models. Such a protocol might accommodate regional variations in drift time. For example, deviations from the original calibration curves might occur quicker in higher concentration applications.

The sensor may have use outside of ambient air monitoring situations including indoor monitoring and in-vehicle monitoring. Recent work by Burnet et al., 2014 has described potential real-world exposures to different types of PM_2.5_ as spanning orders of magnitude in range of concentration. This implies that the maximal response concentration of the sensors must be considered when designing an exposure assessment study. Table 2 presents the maximum detection limit of these sensors for different particles sizes, based on modeling the responses obtained in this laboratory study. Based on these results it may not be appropriate to deploy these sensors in locations with high indoor particle concentration. Because the sensitivity of the sensor decreases as the concentrations approaches the maximum detection limit, distinguishing between levels of exposure at high concentrations may not be possible using this monitor.

Yet, the sensor may still have some use even in high exposure studies, particularly when individuals move in and out of such high exposure situations. In such cases, the sensor might serve as an exposure event “indicator”, noting the frequency and duration of high exposure events. For example, consider the study of Osman et al.[25], who found that the maximum 5-minute average concentration of PM_2.5_ in the homes of smokers (n = 58) in the UK was 491 μg/m^3^ in the maximum 5-minute average concentration in the home of smokers was much higher than the maximum 5-minute average in the home of non-smokers. The Shinyei sensors might be useful for counting smoking events in the home. Another important source on indoor exposure to PM_2.5_ is biomass burning. In Pakistan Siddiqui et al. [26] reported that mean daytime concentration of PM_2.5_ in homes using wood as fuel was 2.74 mg/m^3^_._ The Shinyei sensors could be useful in monitoring biomass cooking and/or heating events but would fail to discriminate between exposure levels in different homes using biomass sources.

We conclude that the sensitivity of this sensor is appropriate for most outdoor locations within the US and Europe, but without modification of the sensor, may not be adequate to capture the full range of higher indoor exposures in homes of smokers or homes where biomass is a source of energy for heat or cooking. It is important to remember that the sensor is a particle counter, and in cases of exposure to polydisperse aerosol of unknown composition, conversion to mass may be difficult without the availability of a concurrent gravimetric sample.

## Supporting Information

S1 Data Underlying data used to generate figures and tables in this paper. Includes the time matched response of the APS and Shinyei sensors for a variety of test atmospheres.
(XLSX)

S1 Fig Size Distribution of ASHRAE dust. (DOCX)

S2 Fig Distribution of the 0.75 μm polystyrene test atmosphere. (DOCX)

S3 Fig Distribution of the 1 μm polystyrene test atmosphere (Large Chamber). (DOCX)

S4 Fig Distribution of the 1 μm polystyrene test atmosphere (Small Chamber). (DOCX)

S5 Fig Distribution of the 3 μm polystyrene test atmosphere. (DOCX)

S6 Fig Distribution of the 6 μm polystyrene test atmosphere. (DOCX)

S7 Fig Percent difference between the mean sensor response and APS values after converting the sensor response to a mass concentration using a linear regression. (DOCX)
