# Correction: Laboratory Evaluation of the Shinyei PPD42NS Low-Cost Particulate Matter Sensor

**DOI:** 10.1371/journal.pone.0141928

**Published:** 2015-10-28

**Authors:** Elena Austin, Igor Novosselov, Edmund Seto, Michael G. Yost

The y-axis label for Fig 5 incorrectly states mean rather than difference. The correct y-axis is “Difference of Shinyei and APS Mass (μg/m^3^)”. The authors have provided a corrected version of Fig 5 here.

**Fig 5:**
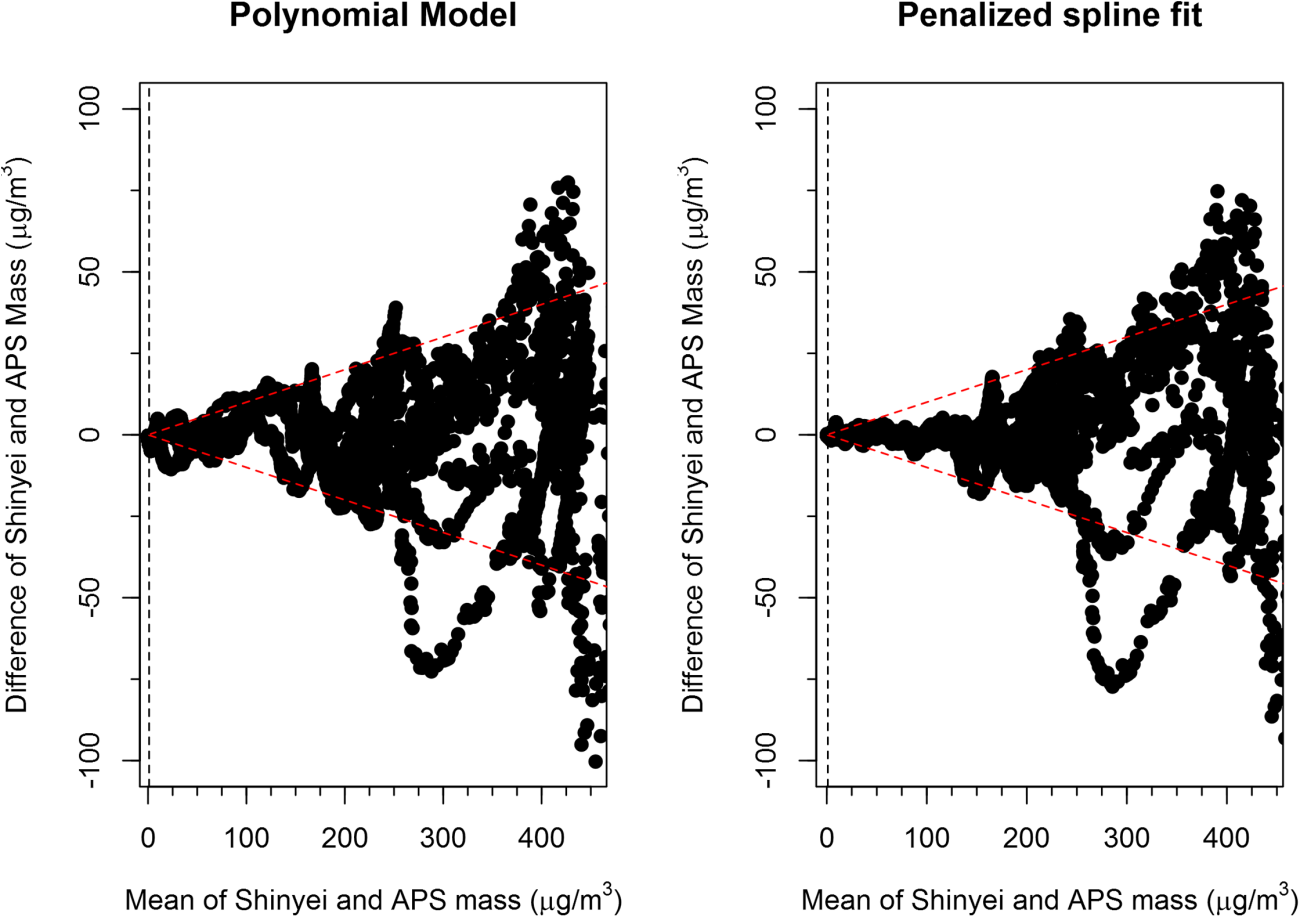
Bland Altman Plots (4 sensors pooled together). The red dashed lines represent a 10% error on the mass measurement. The black vertical dashed line represents the LOD.
