# Supplementary figures and images for: Laboratory Evaluation of the Shinyei PPD42NS Low-Cost Particulate Matter Sensor
Source: PLoS One. 2015 Sep 14;10(9):e0137789. doi: 10.1371/journal.pone.0137789 (PMC4569398; doi:10.1371/journal.pone.0137789)

S1 Fig. Size Distribution of ASHRAE dust


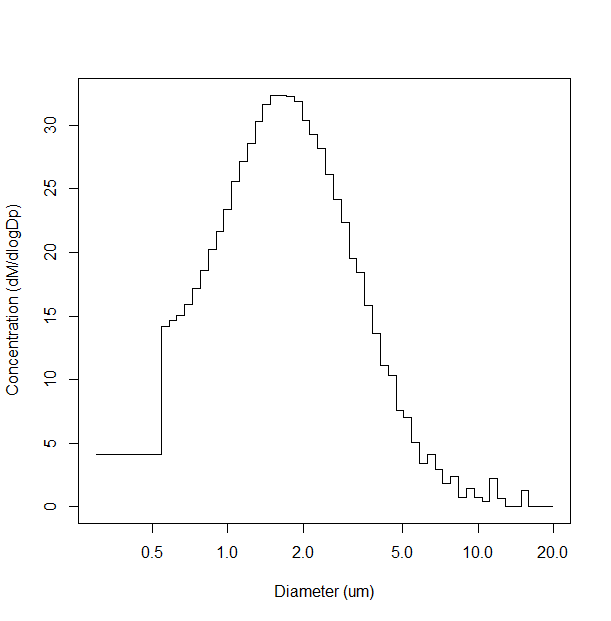


Median diameter of particles: 2.18 µm

Supplement: S1 Fig — (DOCX) [file pone.0137789.s002.docx]

S2 Fig. Distribution of the 0.75 µm polystyrene test atmosphere:


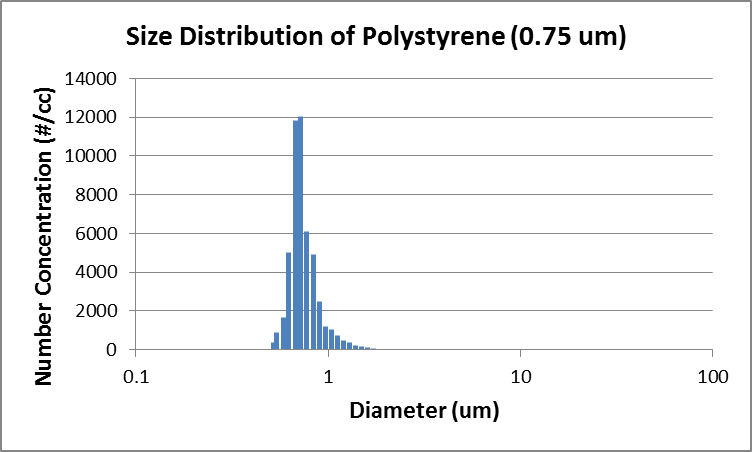


Median diameter of particles: 0.72 µm

Supplement: S2 Fig — (DOCX) [file pone.0137789.s003.docx]

S5 Fig. Distribution of the 3 µm polystyrene test atmosphere:


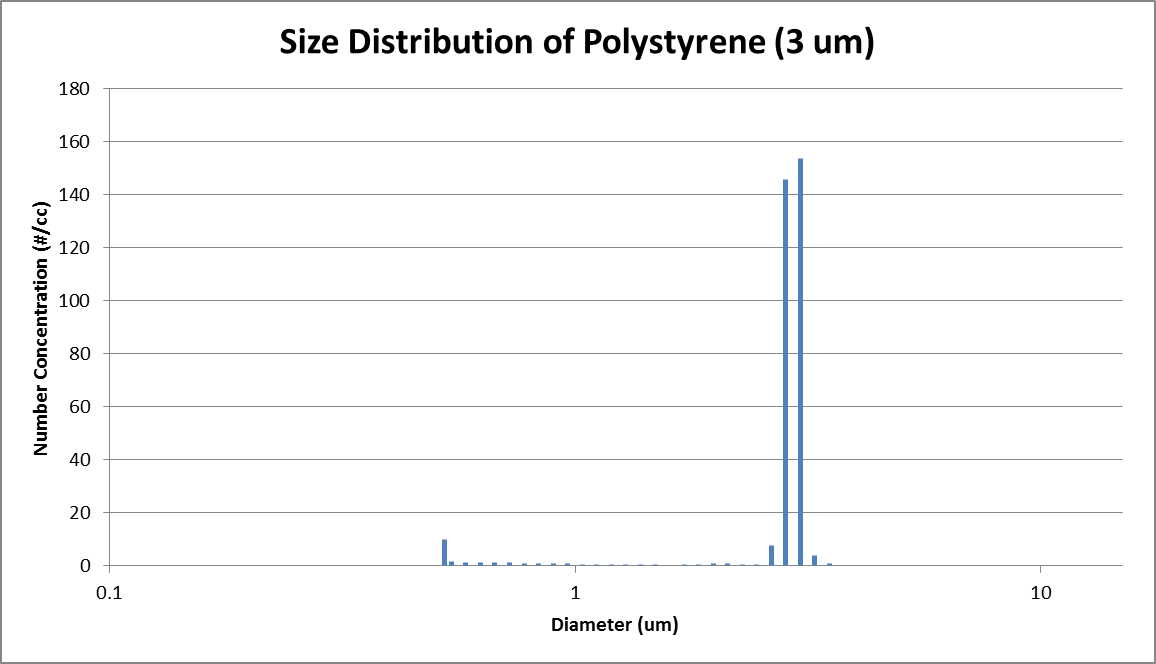


Median diameter of particles: 2.97 µm

Supplement: S5 Fig — (DOCX) [file pone.0137789.s006.docx]

S6 Fig. Distribution of the 6 µm polystyrene test atmosphere:


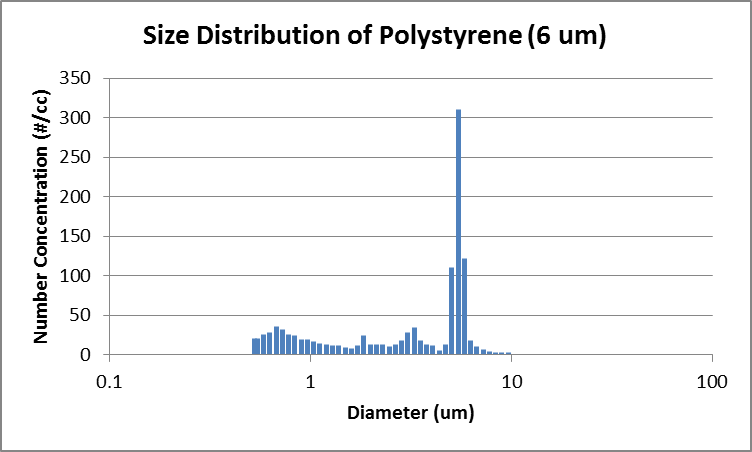


Median diameter of particles: 5.40 µm

Supplement: S6 Fig — (DOCX) [file pone.0137789.s007.docx]
