# Supplementary material for: Laboratory Evaluation of the Shinyei PPD42NS Low-Cost Particulate Matter Sensor
Source: PLoS One. 2015 Sep 14;10(9):e0137789. doi: 10.1371/journal.pone.0137789 (PMC4569398; doi:10.1371/journal.pone.0137789)
Supplement: S4 Fig — (DOCX) [file pone.0137789.s005.docx]

S4 Fig. Distribution of the 1 µm polystyrene test atmosphere (Small Chamber):


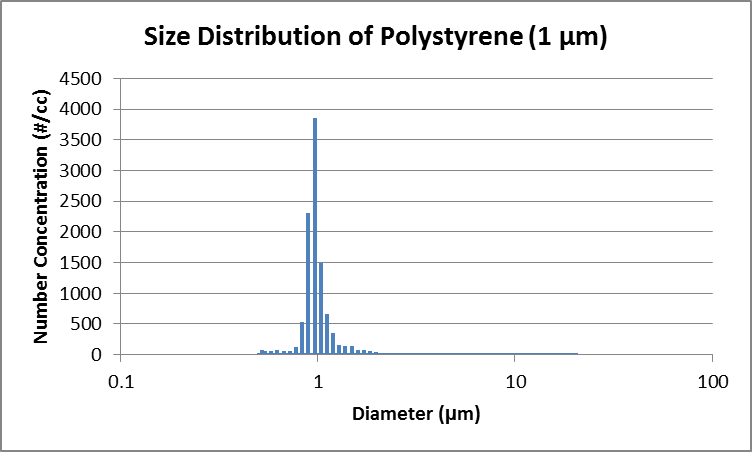


Median diameter of particles: 0.97 µm
