# Supplementary material for: Laboratory Evaluation of the Shinyei PPD42NS Low-Cost Particulate Matter Sensor
Source: PLoS One. 2015 Sep 14;10(9):e0137789. doi: 10.1371/journal.pone.0137789 (PMC4569398; doi:10.1371/journal.pone.0137789)

S7 Fig. Percent difference between the mean sensor response and APS values after converting the sensor response to a mass concentration using a linear regression


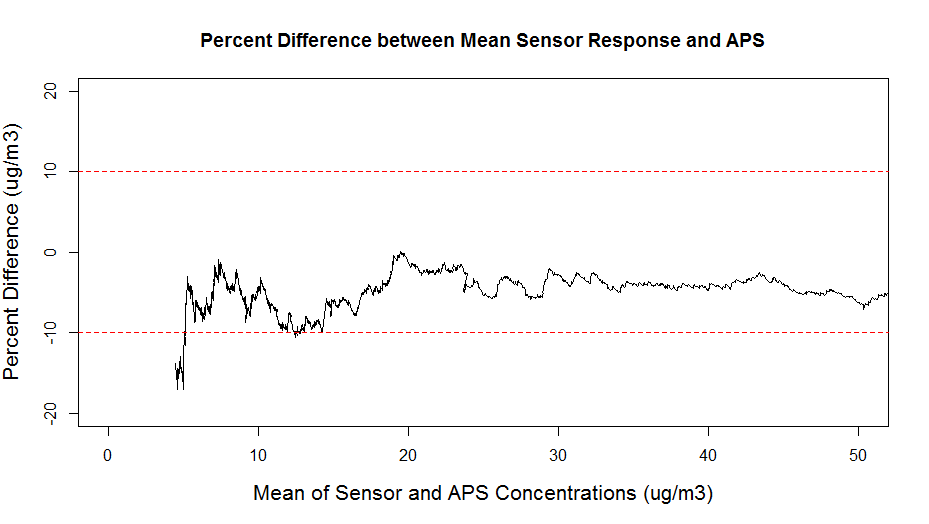

Supplement: S7 Fig — (DOCX) [file pone.0137789.s008.docx]
